# The International Transfer of Patients With Positive SARS-CoV-2 Test Using a Framework

**DOI:** 10.7759/cureus.51336

**Published:** 2023-12-30

**Authors:** Yutaka Furuta, Yoji Hoshina, Yusuke Matsuura, Manami Ueshima, Hiroki Kyo, Tomohiro Arai, Eric Terpstra, Shamis Fallah

**Affiliations:** 1 Pediatrics, Vanderbilt University Medical Center, Nashville, USA; 2 Neurology, University of Utah, Salt Lake City, USA; 3 Pediatrics, Icahn School of Medicine at Mount Sinai, New York, USA; 4 Department of Pediatrics, Kameda Medical Center, Chiba, JPN; 5 MetroAtlanta Ambulance Service, Emory Healthcare Network, Atlanta, USA; 6 Development and Regeneration, Cluster Women and Child, Biomedical Sciences, KU Leuven, Leuven, BEL; 7 Obstetrics and Gynecology, Naval Medical Center Camp Lejeune, Camp Lejeune, USA; 8 Ophthalmology, Navy Medicine Readiness and Training Command Bremerton, Bremerton, USA

**Keywords:** united states naval hospital, public health, pandemic, international transfer, covid-19

## Abstract

The Japanese Fellowship Program at the U.S. Naval Hospital Yokosuka has served as a liaison with Japanese hospitals during the transfer of acutely ill U.S. Navy patients since 1952. The SARS-CoV-2 pandemic has complicated this process and prompted the creation of a new framework that involves the Public Health Center. We present two international transfer cases of patients with positive SARS-CoV-2. The creation of a framework enabled a safe and smooth transfer process of patients with a favorable outcome. This report can help guide future cases of international transfer, especially for patients who need infectious disease surveillance. To our knowledge, we describe the first report of an international transfer of patients with positive SARS-CoV-2 test using a framework.

## Introduction

The U.S. Naval Hospital Yokosuka (USNHY) is one of the U.S. military treatment facilities in mainland Japan (https://www.med.navy.mil/US-NMRTC-Yokosuka-Japan/). USNHY serves military and Department of Defense civilian personnel throughout mainland Japan. While this hospital is located in Japan, the staff of USNHY speak English as do the majority of the patients. Most of the staff is commonly stationed in Japan for two to three years, during which some USNHY staff take Japanese language courses, but few become fluent. Rarely are the USNHY physicians proficient enough to communicate patient information in Japanese. In terms of the hospital capacity, USNHY has 47 beds in total and departments including internal medicine, pediatrics, family medicine, emergency medicine, obstetrics/gynecology, general surgery, anesthesiology, neurology, orthopedics, dermatology, otolaryngology, ophthalmology, urology, psychiatry, radiology, and public health. USNHY does not possess advanced medical resources including magnetic resonance imaging, echocardiogram, cardiac catheterization laboratory, or intensive care units. Thus, acutely ill patients who require a higher level of care need to be transferred to local Japanese medical centers. USNHY has over 180 transfer cases per year on average. However, one of the challenges to a successful transfer between hospitals is communicating the differences in treatment protocols, language barriers, and cultural norms. Among the developed nations, Japan remains one of the most highly homogeneous cultures, or monoculture, as non-Japanese speakers make up only approximately 1.5% of the population. Language and cultural differences may negatively impact an international transfer between two countries [1-3]. To overcome these obstacles, the Japanese National Physician Graduate Medical Education Program (also known as the Japanese Fellowship Program) at the USNHY was established in 1952.

Since 1952, the Fellowship has forged a partnership between Japanese and American healthcare professionals. This fellowship is a one-year program geared to Japanese national board-certified physicians. Six Japanese physicians are annually selected by American medical providers through interviews. One of the critical missions is for Fellows to act as a liaison and a translator with Japanese hospitals during the transfer of acutely ill American patients that exceed the capabilities of USNHY. The liaison role is difficult as it bridges gaps in cultures and standards of care, and it is immensely rewarding to the Fellows and meaningful to the American patients.

Back in 2020, the COVID-19 pandemic caused by SARS-CoV-2 began to spread globally including in Japan [4]. As USNHY was not capable of treating acutely ill patients infected with COVID-19, these patients were required to be transferred to local Japanese medical centers for a higher level of care. In contrast to other disorders, however, a selection process of transfer was authorized by the Public Health Center in Japan. This complicated exempt situation prompted the creation of a new transfer protocol with a framework for COVID-19. To our knowledge, we describe the first report of an international transfer of patients with positive SARS-CoV-2 test using a framework.

## Case presentation

Case one

**Figure 1:**
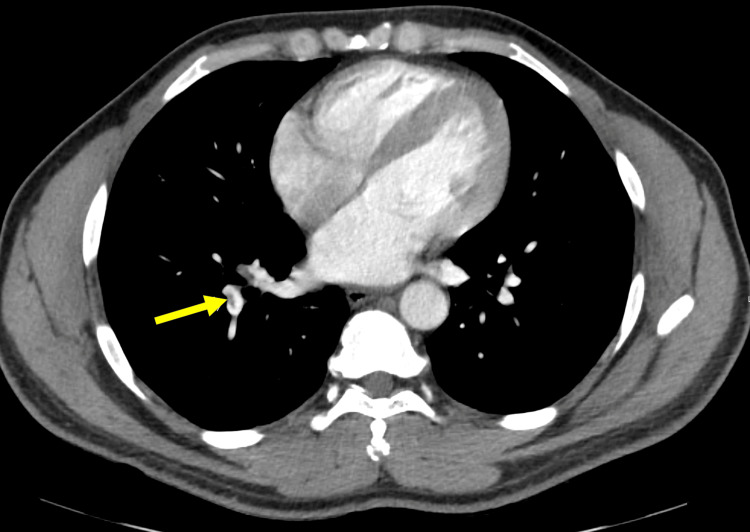
Pulmonary embolism on CT angiogram of the chest (arrow).

A 40-year-old active-duty male with a known COVID-19 infection presented to the emergency department (ED) with palpitations, pre-syncope, fever, congestion, and cough. His screening was found to be positive for COVID-19. The cardiac examination demonstrated tachycardia with normal heart sounds without murmur, rub, or gallop. His respirations were unlabored, and his lungs were clear to auscultation bilaterally. The remainder of the examination was normal. Laboratory findings showed elevated D-dimer of 1,780 ng/mL. Troponin was negative. CT angiogram (CTA) of the chest showed pulmonary embolism (Figure 1) and scattered ground-glass opacity (GGO) consistent with viral infection due to known COVID-19. According to our COVID-19 protocol, our Japanese Fellow Physicians arranged for his transfer to the Japanese hospital to receive higher-level care. He was admitted to the intensive care unit in the local Japanese hospital for further management of pulmonary embolism.

Case two

**Figure 2:**
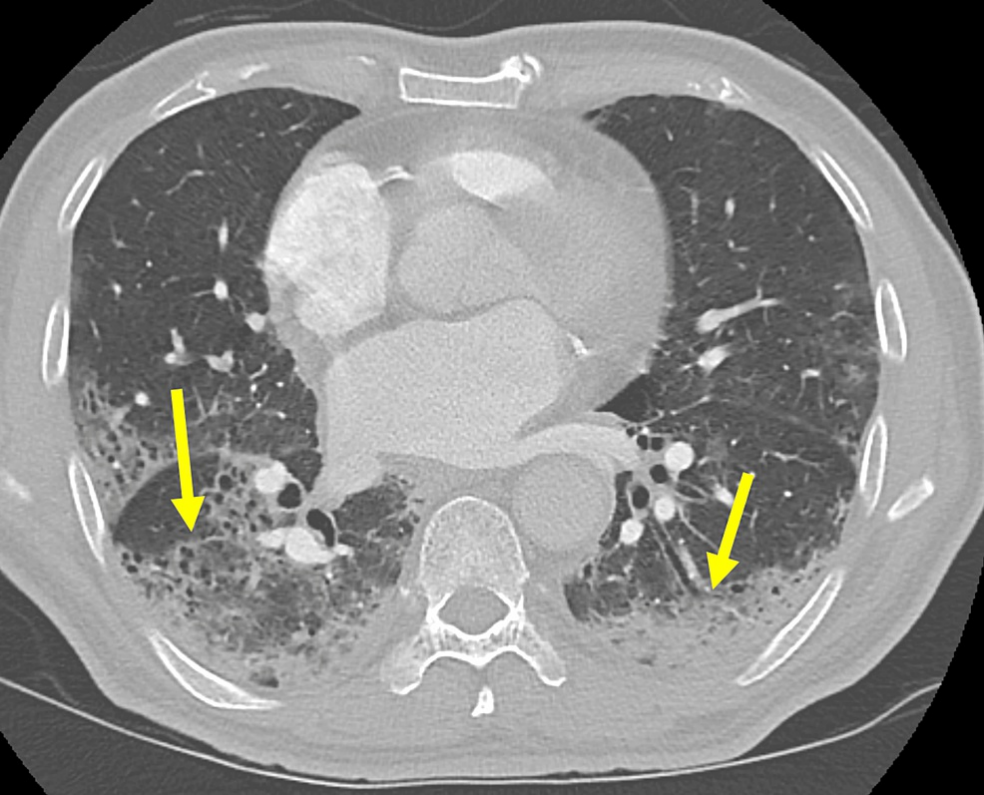
Bilateral peripheral ground-glass opacity and multifocal consolidations within the lungs on CT of the chest (arrows).

A 70-year-old male with a history of hypertension, type 2 diabetes, dilated cardiomyopathy with a last ejection fraction of 19%, and known COVID-19 infection presented to ED with worsening shortness of breath over the past four days. He was diagnosed with COVID-19 infection two days before the presentation. On arrival, his vital signs revealed a heart rate of 83 beats per minute, blood pressure of 119/63 mmHg, respiratory rate of 30 respirations per minute, a temperature of 97.4°F (36.4℃), and an oxygen saturation of 84% in room air. The cardiac examination demonstrated tachycardia with normal heart sounds without murmur, rub, or gallop. His respirations were labored with increased respiratory effort, and his lungs were clear to auscultation bilaterally. The initial electrocardiogram showed ST depressions in the inferior leads. Laboratory findings were significant for elevated troponin of 2.46 ng/mL, lactate of 2.40 mmol/L, and D-dimer of >7,000 ng/mL. CTA of the chest was obtained that demonstrated bilateral peripheral GGO and multifocal pulmonary consolidations (Figure 2). He was transferred to an intensive care unit in the Japanese hospital to receive a higher level of care according to our COVID-19 protocol.

## Discussion

**Figure 3:**
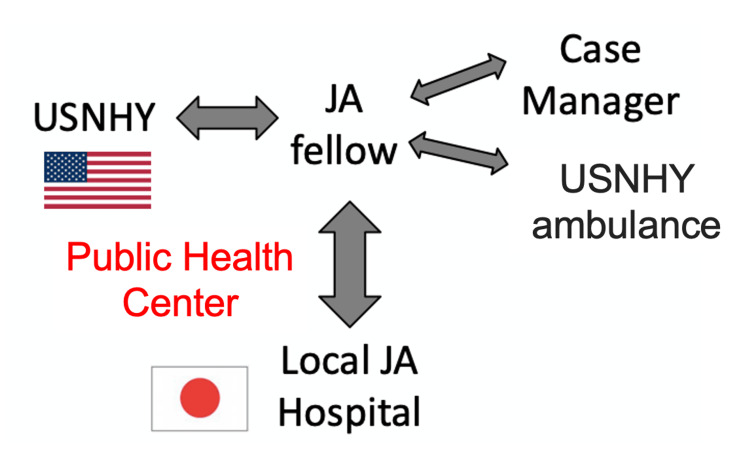
A framework for transferring COVID-19 patients in USNHY. The first step involves USNHY providers formally requesting a transfer through communication with an on-call Japanese (JA) Fellow. Following discussions, Fellows coordinate the transfer by contacting host nation hospitals and identifying an appropriate accepting hospital. Once the accepting hospital is confirmed, transportation is organized using a USNHY ambulance and a case manager is contacted. The patient is then transported under the supervision of a USNHY provider and accompanied by the Fellow. Upon arrival at the host nation hospital, patient care is transitioned to the local Japanese medical staff. USNHY: U.S. Naval Hospital Yokosuka

We describe two cases of patients with COVID-19 and their transfer process between USNHY and the local Japanese medical centers. Our system of transfer is activated when USNHY providers judge to need a higher level of care that USNHY is incapable of. Fellows are a critical and integral part of this transfer process (Figure 3). The initial step of transfer is an announcement of a request for transfer to an on-call Fellow by USNHY providers. After discussion, Fellows arrange a transfer by contacting host nation hospitals and determining an accepting hospital. Once the accepting hospital is determined, transportation by a USNHY ambulance is arranged and a case manager is contacted. Then, the patient is transported under the supervision of the USNHY provider and accompanied by the Fellow. As for the patient transported to the host nation hospital, the patient care is relayed to the Japanese medical staff in the local Japanese hospital.

**Figure 4:**
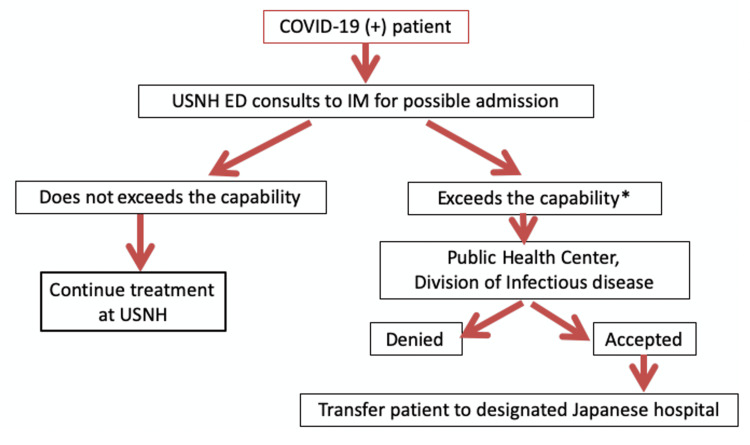
A framework for transferring COVID-19 patients from USNHY. *: Two criteria for transfer: (1) a patient requires elevated oxygen demand, and (2) a patient is at risk of hemodynamic instability. ED: emergency department; IM: internal medicine; USNHY: U.S. Naval Hospital Yokosuka

At the beginning of the COVID-19 outbreak, a shortage in the bed capacity in the local Japanese hospitals made the transfer acceptance more challenging [5]. Thus, to avoid chaotic situations, the selection process of transfer was mediated by the Japanese Public Health Center in Japan. This complicated exempt situation prompted the creation of a new transfer protocol with a prototype for COVID-19 (Figure 4). To our knowledge, we describe the first report of an international transfer of patients with positive SARS-CoV-2 test using a framework. Given our capacity, two criteria for transfer were determined: (1) a patient requires elevated oxygen demand, and (2) a patient is at risk of hemodynamic instability. Once a patient meets either of the criteria, the Public Health Center is contacted by Fellows to determine an accepting hospital instead of a direct contact to local Japanese hospitals as it had been the case for non-COVID-19 transfers. Then, the Public Health Center designates an appropriate accepting hospital. The involvement with the Public Health Center helped to secure beds for patients with COVID-19 and transfer the patients in a timely and secure manner. This process involves on-call Japanese infectious disease experts, including physicians in the Public Health Center, who clinically assess patients’ conditions and determine the appropriate accepting hospital. If unable to secure an accepting hospital, patient care is expected to either continue at USNHY or consider medical evacuation to the United States, referred to Medevac. Fortunately, we have not encountered any instances of failing to secure an accepting hospital. Without cooperation of the Public Health Center, it is certain that it would take longer to locate and receive approval from an accepting hospital in the setting where the capacity of majority of the hospitals were exceeded.

The first patient presented with pulmonary embolism and the second one was a high-risk elderly patient who exhibited acute respiratory distress due to pneumonia, both of whom required a higher level of care. Both patients met the transfer criteria in terms of an oxygen demand and a high risk of hemodynamic instability. The transfers of these two cases were conducted safely and smoothly based on our framework was established in advance. The creation of a framework enabled a safe and smooth transfer process of patients leading to a favorable outcome. Although COVID-19 may no longer be a global health emergency, we conclude this report may help guide future cases of international transfer, especially for patients who need infectious disease surveillance.

## Conclusions

The creation of a framework for the international transfer of COVID-19 patients facilitated a secure and smooth transfer, resulting in a positive outcome. This report can serve as a valuable guide for future cases of international transfer, particularly for cases requiring infectious disease surveillance.
